# The role of community health workers in strengthening health system resilience in Somalia

**DOI:** 10.1186/s41182-026-00964-0

**Published:** 2026-05-26

**Authors:** Liibaan Abdulahi Sudi, Yakub Burhan Abdullahi, Mohamed Daud Mohamed, Abdikadir Ahmed Hassan, Iqro Omar Ibrahim, Abdirahman Mohamed Adan

**Affiliations:** 1 Faculty of Health Sciences and Tropical Medicine, Somali National University, Mogadishu, Somalia; 2 Faculty of Health Sciences, Salaam University, Mogadishu, Somalia; 3 Center for Health Research and Innovation, Somali National University, Mogadishu, Somalia; 4 Mogadishu Institute of Health, Mogadishu, Somalia

**Keywords:** Community health workers, Health system resilience, Somalia, Fragile settings, Primary health care

## Abstract

Community health workers (CHWs) may play an important role in strengthening health system resilience in fragile and conflict-affected settings. Global evidence suggests that CHWs can improve maternal and child health outcomes and expand access to essential care in low-resource contexts. In Somalia, where conflict, disease outbreaks, and workforce shortages constrain health service delivery, CHWs appear well-positioned to bridge gaps between communities and formal systems. Evidence from Somalia’s COVID-19 response indicates that CHWs identified approximately one-third of suspected cases and demonstrated higher case positivity rates than facility-based surveillance. CHWs have also contributed to maternal health service uptake in underserved areas. However, Somalia-specific evidence remains limited and context-dependent. Current CHW programs face challenges including inconsistent integration into national systems, variable supervision, and reliance on fragmented external support. Strengthening CHW contributions may require formal recognition within national health service frameworks, sustainable financing, standardized training protocols, and improved supervision mechanisms. While CHWs alone cannot resolve systemic health challenges, evidence suggests that deliberate investment in their capacity and integration could support broader health system resilience efforts in Somalia.

**Dear editor,**

Somalia’s health system operates within one of the world’s most challenging environments, shaped by protracted conflict, recurrent disease outbreaks, climate-induced displacement, acute workforce shortages, and decimated infrastructure [1]. In this context, community health workers (CHWs) are not merely supplementary actors filling gaps in formal service delivery; they play an important role in supporting health system resilience.

Global evidence consistently demonstrates that CHWs are effective in improving maternal and child health outcomes in low- and middle-income countries, particularly in the delivery of preventive interventions such as breastfeeding promotion, essential newborn care, and malaria prevention [2]. In conflict-affected and fragile settings specifically, a systematic review of 55 studies across 19 countries found that CHW-delivered interventions are not only effective but efficient in circumventing access barriers, leveraging physical proximity and social connection to communities to strengthen disease detection, improve care-seeking, and enhance treatment adherence [3]. These findings hold particular relevance for Somalia, where large segments of the population, including nomadic and internally displaced communities, remain beyond the reach of facility-based services.

Somalia-specific evidence, although still limited, suggests that CHWs can make important contributions in selected settings. During the first year of the COVID-19 pandemic, community-based surveillance involving CHWs identified a substantial share of suspected cases and contacts, indicating their potential value for surveillance in hard-to-reach areas [4]. In addition, reports from the Marwo Caafimaad program suggest improvements in maternal service uptake in some underserved districts. However, these findings should be interpreted within the context of program-specific implementation and do not necessarily reflect uniform performance across all regions of Somalia. Furthermore, the Marwo Caafimaad CHW program has been associated with measurable improvements in antenatal care coverage and skilled birth attendance, particularly in rural districts, with qualitative evidence highlighting the critical role of culturally aligned female health workers in bridging sociocultural barriers and building community trust in the health system [5].

Despite this evidence, CHWs in Somalia remain largely unintegrated into formal health system structures, inadequately financed, and inconsistently supervised. This represents both a policy failure and a missed opportunity. Priority actions could include: (1) formally defining CHW roles within the Essential Package of Health Services, including referral, health promotion, and community surveillance functions; (2) establishing a standardized national training and certification framework with regular refresher training; and (3) developing a supervision and reporting model linked to routine district health systems, including digital reporting platforms where feasible. In parallel, government and partners should work toward a more predictable financing model for CHW programs to reduce reliance on fragmented short-term projects.

In Somalia, CHWs appear to be among the most accessible community-level health actors, particularly where facility-based services are limited. Investing in this workforce may be an important component of efforts to strengthen health system resilience in Somalia.

This correspondence is based on published global evidence and selected Somalia-specific reports. The Somalia evidence base remains limited, context-specific, and insufficient to support broad causal conclusions about national CHW performance across all regions and services.

## Data Availability

No datasets were generated or analyzed during the current study.

## Abbreviations

**CHWs**: Community health workers
**DHIS2**: District health information software 2
